# Studying Children’s Eating at Home: Using Synchronous Videoconference Sessions to Adapt to COVID-19 and Beyond

**DOI:** 10.3389/fpsyg.2021.703373

**Published:** 2021-07-22

**Authors:** Shruthi Venkatesh, Jasmine M. DeJesus

**Affiliations:** Department of Psychology, University of North Carolina at Greensboro, Greensboro, NC, United States

**Keywords:** meal observation, children’s eating behavior, online research, food preferences, food intake

## Abstract

The COVID-19 pandemic has disrupted many facets of developmental research, including research that measures children’s eating behavior. Here, children’s food intake is often measured by weighing foods that children are offered before and after in-person testing sessions. Many studies also examine children’s food ratings (the extent to which they like or dislike a food), assessed *via* picture categorization tasks or hedonic scales. This paper reviews existing research on different methods for characterizing children’s eating behavior (with a focus on food intake, preferences, and concepts) and presents a feasibility study that examined whether children’s eating behaviors at home (including their food intake and ratings) can be measured *via* live video-chat sessions. The feasibility analyses revealed that an observational feeding paradigm at home yielded a majority (more than 70%) of video-chat recordings that had a sufficient view of the child and adequate sound and picture quality required for observational coding for the majority of the session’s duration. Such positioning would enable behavioral coding of child food intake, parent food talk, and meal characteristics. Moreover, children were able to answer questions to stories and express their preferences *via* researcher screen-share methods (which can assess children’s self-reported food preferences and beliefs) with low rates of exclusion across studies. The article ends with a discussion on the opportunities and challenges of using online platforms to conduct studies on children’s eating behaviors in their home environments during the COVID-19 pandemic and beyond.

## Studying Children’s Eating at Home: Adapting to COVID-19 and Beyond

COVID-19 has upended many aspects of the research process (not to mention the lives of researchers and the families we study). Before the pandemic, researchers ascertained the validity of remotely collecting data from children of a variety of ages using asynchronous measures, including webcam recorders for looking-time paradigms with infants (Semmelmann et al., 2017; Tran et al., 2017) and unmoderated research platforms, such as LookIt (Scott and Schulz, 2017) and Discoveries in Action (Rhodes et al., 2020). These platforms allow children to complete studies without interacting with a researcher directly (but with some assistance from the parent or guardian providing consent). Many of these methods have been recommended during the pandemic to continue and potentially improve data collection into the future (Sheskin et al., 2020). Synchronous methods of remote data collection, such as Zoom, have also become popular as they allow researchers to interact with and collect data from families in live time (Kuo et al., 2021). However, there is limited work on the feasibility of studying children’s eating behavior (a key line of research in our laboratory) using remote online research tools. In this paper, we document the successes and challenges we have experienced in adapting our research using online methods. In the upcoming sections, we highlight previous work that has measured infants’ and children’s eating behavior using the amount of food eaten and food preferences or concepts as outcome measures in the laboratory or outside the laboratory in home or school settings. We present data from a feasibility study that examined children’s typical meals at home and food preferences *via* live video-chat sessions. We conclude with a discussion on the opportunities to ask innovative questions about children’s eating behavior at home during and after the COVID-19 pandemic using such online platforms.

## Measuring Children’s Food Intake

### Comparing Pre- and Post-test Food Weight

Prior to the COVID-19 pandemic, researchers interested in examining children’s food intake took a variety of approaches in measuring what and how much children ate during a research study. A common and intuitive approach to this practice was to weigh a food that children were offered before the study and weigh that food again after the study as a measure of how much children ate. In a comprehensive review on experimental studies that seek to change children’s eating behavior, 29 of the 120 studies reviewed used weighed food intake as a dependent variable (among other common outcomes, such as food preferences or choices which will be described in the upcoming sections), specifically for studies that sought to increase children’s fruit and vegetable intake (see DeCosta et al., 2017 for review). Many of our own studies take this approach, including studies that examine how social knowledge of the food influences children’s food intake (DeJesus et al., 2018b), whether children eat more food if they assisted in preparing the food (DeJesus et al., 2019a), how maternal talk and intake of food relates to children’s intake of those foods (DeJesus et al., 2018a), and whether children learn about food by verbal testimony or by seeing someone eat that food (DeJesus and Venkatesh, 2020). When this in-person interaction is not possible, it is harder for researchers to use pre-post weight measurements as a standardized measure of food intake given the access to and variability of weighing scales that families may have at home.

### Measuring Food Intake *via* Bites or Pieces of Food Eaten

In addition to measuring intake based on food weight, researchers can code the number of bites (solid intake) of food taken during feeding sessions which can be coded from video recordings. For older infants and children who eat solid foods, food bites as an outcome variable are indexed by coding for every time the food passes through the children’s lips. As an example, to validate maternal reports of their child’s selective eating against children’s observed food intake, Fernandez et al. (2018) examined data from an observational paradigm during which familiar and unfamiliar foods were offered. Researchers measured the children’s latency to their first bite of food and the total number of bites in the videos by counting the number of times the food passed through the infant’s lips in 10-s intervals (Fernandez et al., 2018). Similarly, in a study examining one-year old infants’ temperament and feeding history as predictors of their receptivity to unfamiliar foods, infant’s acceptance of the food was coded from videos in 5-s intervals (Moding et al., 2014). Here, acceptance was defined by when the infants opened their mouths in anticipation of the next bite, smiled and reached toward the food, or the food successfully passed through their lips. Food rejection was coded when the infants physically removed the foods from their mouths, fussed, or turned their mouths away. Intake in bites can also be captured in terms of children’s choice of one food over another (e.g., do children take their first bite of food A or food B?), where the foods that the infants reach toward and taste first are measured (Shutts et al., 2009). Thus, food bites can be one avenue through which researchers can gather quantitative information on food intake, and we aimed at exploring whether such data can be collected through online data collection methods.

Another quantitative measure of food intake is counting the number of discrete pieces of food eaten. For example, if a child is offered 10 carrot sticks, how many carrot sticks did the child eat? In an intervention that sought to conceptually explain food as a source of nutrition to preschool children, researchers live coded children’s snack intake during snack time at their preschool setting (Gripshover and Markman, 2013). The authors found an increased intake of vegetables post the intervention in children; here, the number of pieces of snack consumed was measured by number of pieces of food chosen minus those left after the snack time (such as crackers and vegetables). Comparably, to test the IKEA effect, or the idea that people prefer self-crafted products over similar products made by others (Norton et al., 2012), in children, Raghoebar et al. (2017) explored whether children would consume more vegetables if they created the snack themselves. Children crafted a peacock out of either snack vegetables or colored beads and their vegetable intake was measured by the number of vegetable pieces (e.g., cucumber) pre-post intake.

An extension of this method to examine food choices is to assess children’s choices when the same food is presented in different conditions. To investigate whether the knowledge that a food is healthy or can help with an intellectual goal will imply that the food tastes less good, 3- to 5.5-year-old children were offered either crackers or carrots across five experiments. Based on their condition, they received “healthy,” “yummy,” and control (no message) messages of the food, with the amount eaten (in terms of pieces), the number of pieces of food chosen to take home, and perceived ratings of the food as the dependent variables (Maimaran and Fishbach, 2014). Such coding eliminates the added personnel power, software, and time needed for coding bites as described previously and can be completed live during the testing session. However, the number of foods that can be counted as discrete pieces is restricted in comparison with the variety of textures and forms of food infants and children consume in their home environments.

## Measuring Food Preferences and Concepts

In addition to actual food intake, children’s food preferences can also be assessed, either in addition to their food intake or as a primary outcome without offering children real foods. Such studies typically highlight children’s understanding of food groups, their own food preferences, and their other beliefs about food, such as potential connections between food and cultural groups. Children’s verbal attestation of their food preferences, likes and dislikes can be measured through picture choices, brief scale ratings, and sorting tasks. Children can be asked to report their preference on a scale through smiley face rating scales (ranging from “not yummy at all” to “really really yummy” or “dislike” to “like;” Zeinstra et al., 2010; DeJesus et al., 2018b), a series of questions, such as “Is [name of food] yummy or yucky? Really (yummy/yucky) or a little (yummy/yucky)?” (DeJesus et al., 2019b), or as a choice between two options (Echelbarger et al., 2020). For preverbal infants who cannot say if they like a food or not, a few methods are still available to assess their preferences or early reasoning about food: infants’ facial expressions or parent ratings can provide some insight into their food enjoyment (Mennella et al., 2001).

Similar methods can be used to understand children’s thinking about other aspects of food, such as their social relevance and taxonomic categories. For instance, when presented with pictures of foods that included conventional and unconventional combinations, in addition to their own preferences, children’s social judgments about people who ate those foods were assessed with questions, such as “do you want to be friends with [name of person who eats that food] or not really?” (DeJesus et al., 2019b). Social judgments can even be assessed in preverbal infants using looking-time paradigms, such as examining whether people who share a food preference are especially likely to socially affiliate (Liberman et al., 2016). Finally, card sorting tasks have been used to examine children’s ability of food categorization as a precursor to food rejection (Rioux et al., 2016). Here, children were shown pictures of fruits and vegetables that varied in color, typicality, and whether the foods had been cubed or sliced. Children completed tasks, such as sorting those pictures into categories, naming the colors of the fruits and vegetables, and discarding foods they were unwilling to try (Rioux et al., 2016). In these ways, researchers can assess infants and children’s food preferences and ratings verbally and nonverbally, through picture choices, brief scale ratings, sorting tasks, and looking time paradigms. In this paper, we hoped to examine the feasibility of collecting children’s self-reported preferences *via* an online format.

## Parental Reports of Children’s Food Intake

Parental recall and reports of their children’s diet can provide descriptive data on what kinds of food their children eat, which can be standalone data and predictors or outcomes in studies that also have meal observations. In a study that combined naturalistic home meal recordings with parental report data, parents reported on their toddlers’ food intake *via* three 24-h dietary recall interviews, and the foods stated were later coded into food groups, specifically fruits and vegetables (Edelson et al., 2016). Videos of meals at home over a day were collected and children’s acceptance or refusal of the foods were coded, along with parental food talk language (prompts). Among other findings, the more fruit and vegetable prompts parents used during the recorded meals, the more parents reported their child ate fruits and vegetables in a 24-h dietary recall task (Edelson et al., 2016). While parent recall was used as an outcome variable in this study, such reports can also be used as predictor variables. Indeed, in a longitudinal study examining infant growth trajectories, mothers reported on their infants’ food frequency and milk (breast milk, formula, or other milks) intake across 7 days as a predictor of child obesity at 6 years (measured by BMI) with infants’ change in weight-for-length z-scores over the first year post-partum as the mediator of this relationship (Ventura et al., 2020).

In addition to parent dietary recall, parent reports on their children’s eating habits and dietary patterns are another common source of data. As an example, the Child Eating Behavior Questionnaire developed by Wardle et al. (2001) consists of subscales, such as food responsiveness, children’s food fussiness, children’s emotional over and undereating, food enjoyment, desire to drink, and satiety responses. This scale of parent report that can be used to predict children’s obesity has been validated against behavioral measures of children’s obesogenic behaviors (Carnell and Wardle, 2007). Furthermore, the Comprehensive Feeding Practices Questionnaire is another commonly used parent-report measure that contains 12 subscales of feeding practices, such as using food as a reward, routine of eating, and teaching nutrition (Musher-Eizenman and Holub, 2007). This questionnaire can be administered *via* paper-pencil or online survey, which lends its flexibility for being used in different settings. In these ways, parents can not only provide data on their children’s eating behaviors, but can also help in collecting such data *via* online formats, which will be elucidated in our Methods and Discussion.

## The Present Study: Feasibility of Measuring Food Intake and Ratings Online

Prior research provides multiple methods to study children’s eating behavior, including naturalistic video recordings that capture children’s eating at home. However, there is a dearth of research that analyzes the validity and plausibility of adapting these measures to remotely study children’s eating behavior using synchronous videoconference sessions. The COVID-19 pandemic has disrupted our ability to invite families into the laboratory for a feeding experimental study or even manage the personnel required for home video recordings. With the shift of our field toward remote online data collection over the course of this past year, our laboratory also transitioned to collecting data from children and families through synchronous videoconference sessions as we describe in two methods. In Method 1, we describe the online remote methods to observe children’s typical meal times at home, and the likelihood of being able to code certain behaviors from these video recordings, such as whether coders could see the children’s face and mouth and hear the parent’s talk during the session. In Method 2, we describe a synchronous videoconference method that could be used to attain children’s food ratings, categorizations, or other aspects of their reasoning about food.

## Method 1

### Observations of Eating at Home

Video recordings of young children’s meals at home have yielded information about the characteristics of the family meal and parental food talk (Bergmeier et al., 2015b). Moreover, videos have also been a method through which their actual food intake has been coded, by measuring liquid sucking, food bites, and behaviors related to acceptance or rejection of foods (Lumeng et al., 2007; Moding et al., 2014; Fernandez et al., 2018). The goal of this current study was twofold. First, we aimed at collecting pilot data to examine typical meals at home for children under 3 years of age and assess the feasibility of conducting these studies using synchronous videoconference sessions. Second, we hoped to test the plausibility of conducting an experimental manipulation of feeding behaviors in an environment naturalistic to the child, which could be an externally valid approach even beyond the COVID-19 pandemic.

### Participants

Children under the age of 3 years were recruited for this study. Participants were recruited from an existing database of volunteer families, social media advertising, and Children Helping Science, an online platform for researchers to advertise online studies and for parents to sign up for studies. Parents were informed *via* email that we would like to set up a half-hour videoconference during their child’s typical meal or snack time, and the appointment was scheduled accordingly (parents were given the flexibility to choose what meal was observed). We were predominantly interested in testing infants as they transitioned to solid foods and toddlers as they expanded their repertoire of solid foods, which is why this age range was chosen. We also aimed to offer an activity for younger siblings of children participating in other research projects designed for children aged 3 years and older.

We had 50 children (25 females, *M_age_* = 17.88 months, *Range_age_* = 0–55 months) participate in the study, with three sibling pairs who participated in the same session together. Though the target age for this study was 3 years and under, one child in the sibling pair was 4 years old and was eating a meal along with their younger sibling. Since this was a typical setup for the family, the older child’s data were retained. Parents identified the majority of our child sample as not Hispanic/Latino (47 or 94%) and as Caucasian/White (42 or 84%; see Table 1). Parental demographics indicate that 36 (72%) parents had graduate degrees, and 26 (52%) reported combined annual household income to be more than $120,000 (see Tables 2 and 3). All parents reported English as a language spoken at home, and 14 (28%) reported a secondary language (such as Russian or French). Since this is a feasibility study, we sought to retain all participant videos to document the range and frequency of issues that would potentially hinder behavioral coding. However, we had decided to exclude videos if they were so poor in quality that even the feasibility analysis (described under “Descriptive Data of the Feeding Sessions”) could not be extracted from these videos. Our other exclusionary criteria included if children were distressed by the presence of the video recording device. None of the sessions fit these criteria, hence, we did not exclude any video recordings.

**Table 1:** Child racial and ethnic distribution (Method 1–2).

	Method 1	Method 2
	(*n* = 50)	(*n* = 181)
Latinx	2 (4%)	13 (7%)
Caucasian/White	40 (80%)	104 (57%)
African-American	1 (2%)	10 (6%)
Asian/Asian-American	1 (2%)	32 (18%)
More than one race	5 (10%)	9 (5%)
Prefer not to respond/no response	1 (2%)	13 (7%)

**Table 2:** Primary parent education (Method 1).

	Frequency
High school/GED	1 (2%)
Associate’s degree	3 (6%)
Bachelor’s degree	7 (14%)
Some graduate school	2 (4%)
Graduate/professional degree	36 (72%)
Other	1 (2%)

**Table 3:** Combined household income (Method 1).

	Frequency
Less than $15,000	2 (4%)
$25,000–$40,000	2 (4%)
$40,000–$60,000	6 (12%)
$60,000–$90,000	5 (10%)
$90,000–$120,000	5 (10%)
More than $120,000	26 (52%)
Prefer not to respond	4 (8%)

### Materials and Procedure

Once the videoconference appointment was scheduled, parents were emailed an online consent form. This form also included a media consent form which gave us permission to videotape this interaction and potentially use the audio and video recordings (such as at conferences, for teaching materials, or on our laboratory Web site). All parents consented to being recorded, though there was variability in the permissions granted for the use of these recordings (see Table 4). Parents reported on demographics, such as their race, ethnicity, educational attainment, household income, and languages spoken at home.

**Table 4:** Parent media permission (Method 1).

	Yes	No	Missing
Showing videos, audio, or images in the classroom	42 (84%)	7 (14%)	1 (2%)
Showing videos, audio, or images in academic meetings or conferences	40 (80%)	9 (18%)	1 (2%)
Showing videos, audio, or images on our laboratory Web site	24 (48%)	25 (50%)	1 (2%)
Including images in publications of this study and on online repositories, such as the Open Science Framework	27 (54%)	22 (44%)	1 (2%)
Including images in newsletter we send to families interested in our research	28 (56%)	21 (42%)	1 (2%)
Including images in promotional materials (such as brochures or flyers)	27 (54%)	22 (44%)	1 (2%)

In this email, parents were also sent a guide to help navigate them through the video-chat platform if needed (full text available on the Open Science Framework).^1^ This guide contained screenshots for how participants could join the meeting and turn on their video and audio settings. We used WebEx when our online data collection began in May 2020 as our institution’s IRB had already approved research studies using that platform. By October, we learned that our university would be ending its subscription with WebEx and we transitioned to using Zoom for data collection. Zoom was also more familiar to parents (a few parents asked if we could use Zoom instead) and was an easier platform to use (though we did not experience any technical difficulties that resulted in participant exclusion specifically because of difficulties with the WebEx platform).

Researchers conducted the study on a university-issued laptop or desktop device. Parents typically logged in from their laptops, but they also could log in from their tablet, phone, or desktop computer. At the start of videoconference session, the researcher introduced the study to the parent and started recording the session. The recording was done directly to the device the researcher was logged in on and not on the WebEx/Zoom cloud for participant privacy. Parents were asked some questions before the start of the feeding session regarding what their child was going to eat, if the child would be sitting in their typical seat, how often they had been introducing new foods to their child during the pandemic, and if there was anything about the current pandemic situation they would like to share (*see OSF for full text*). Once they were ready to start with the feeding session, the researcher suggested that parents could cover their screen with a sheet of paper (without covering the camera), or swipe to another application on their device if the child seemed distracted by seeing themselves eat or if eating in front of a screen was atypical for them. If the parents chose to do this, it was ensured that the camera view of the feeding setting was not blocked. The researcher then told the parents to “do what you would usually do as if we were not there” and told the parent they would return if the parent said they were done with the session or after 30 min had passed. The researcher did not provide any additional setup instructions to the parents, as the goal was to assess the quality of the videos that could be recorded with minimal researcher guidance. The researcher then muted/turned off their video and started a 30-min timer.

During the videoconference session, the researcher made live notes of some characteristics of the feeding session, such as if the parent–child dyad was in the frame, if the food was visible, whether it was an individual or family meal, and whether the child was self-feeding or being fed (*see OSF for full text*). After 30 min passed or the researcher heard the parent say, “we are done” (whichever came first), the researcher then turned their video back on and unmuted, and to conclude, asked the parent whether they noticed any differences from a typical meal and if there was anything else they thought would be important for us to know. The child was emailed a certificate and an age-appropriate e-story book from the “Amazing Books for Children” series by the Center for Disease Control and Prevention.^2^ The recorded video was then uploaded to our laboratory’s secure Box folder. This study and the study described under Method 2 were conducted in 2020–21 and approved by the University’s Institutional Review Board (20–0365, “Online child development studies”). Deidentified data and relevant research materials are available on the Open Science Framework (see footnote 1).

#### Video Issues Coding

The goal of this study was to document the feasibility of assessing children’s feeding behaviors *via* recordings of synchronous video-chat sessions. Specifically, we intended to illustrate the plausibility of coding child food intake in bites and parental speech and behavior during meals. To this end, we developed a coding scheme to record potential issues in these video recordings, or a characteristic in the recorded feeding time that would interfere with our behavioral coding goals. We identified 10 types of issues that could appear in these video recordings: (1) cannot see parent’s face (2) cannot see child’s face (3) parent’s hand comes in front of child’s mouth (4) video too dark (5) audio not clear (6) child’s mouth blocked during bottle feeding (7) cannot see individual children (when more than one child participated at once) (8) child moves in and out of frame (9) some speech not in English, and (10) Internet connectivity issues (*see OSF for full text*).

First, we stated if each of these issues was present in the video or not. If it was present, then we quantified the severity of the issue, or for how long in the feeding session the issue occurred. For example, if a researcher intended to code maternal engagement with the child during the feeding session, and the mother was in the frame for most of the video but stepped out of the frame for a few minutes to refill the child’s plate, the coding would still be possible for most of the session. In contrast, if the child was sitting in front of a window and was backlit for the whole meal, then it would be harder to code their food intake or parent–child engagement.

For each issue, we coded whether it occurred for the whole video (100%), most of the video (75%), about half the video (50%), little of the video (25%), or not at all (i.e., it was not an issue in the video). These degrees of severity were estimated based on the duration of the feeding session. For instance, if a feeding session was about 20-min long, we noted first if the issue occurred or not. If it did occur, then we saw whether it occurred for little of the video (5 min), half the video (10 min), most of the video (15 min), or the whole time (20 min). For brightness of the video, we added an additional code “can still see child and food set-up, but brightness is not great” as a comparison for videos that were very clear in terms of visibility to those that were less clear. For bottle feeding and parent language, we coded the presence of these issues given the proportion of time that the behavior occurred. For instance, the mother could be talking for the whole duration to other family members in addition to the child. We coded the language the mother talked to the child in and, if bilingual, assessed the proportion of time the mother did not speak in English to the child. Similarly, if children had bottle feeds (milk/water) during their solid food sessions, for example, they drank out of a bottle for 3 min of a 15-min meal session, then we coded whether their mouth was blocked or not during those 3 min. A team of four coders established inter-rater reliability for 20% of the dataset and had inter-class Kappas of at least 0.76 for each code.

### Results

#### Descriptive Data of the Feeding Sessions

One parent participated when their infant was bottle fed at 3 months, and again 4 months later when the infant had transitioned to solid foods. For the analysis to follow, we included both their videos as a measure of bottle and solid feeds. Seven parents scheduled a session and filled out the consent form but did not attend or reschedule the appointment. Of the 48 videos (*n =* 44 individual child sessions, *n* = 1 child repeated at two time points, *n* = 3 sibling sessions), the mother attended the appointment for 41 sessions (85%), the father attended the appointment for three sessions (6%), and both parents attended the appointment for four sessions (8%). 33 sessions (69%) were individual meals where only the child was eating, while 15 (31%) were family meals, where we could see the child as well as other family members eating a meal. Furthermore, eight (16%) children were fed by the parent, 28 (55%) children self-fed, and 15 (30%) had a mix of both, self-feeding and being fed.

In terms of the type of feeding involved, three (6%) were only bottle feeds, while the majority (48 or 94%) was solid food sessions. 17 feeding sessions (35%) lasted the whole 30 min. From the sessions that did not last for 30 min (i.e., sessions that ended because the parent said they were done), 22 min was the average duration of the meal.

#### Parent Interview

With regards to the parents’ description of the meal, all children sat in their typical seats during the meal. Since the start of the pandemic, 15 parents (30%) said they have been introducing new foods to their child more than usual, two (4%) less than usual, and 26 (51%) about the same pace as before. 27 (53%) parents described the session as representative of a typical meal. Some common responses for atypicality of the meal were “Normally my husband and I will talk to each other more during breakfast” or “we usually start with a food he [the baby] likes and then offer a new food, but we thought you would be interested in seeing him eat a new food so we started with that first.”

#### Video Issues Coding

Child visibility. A majority of the videos did not contain issues that would potentially hinder behavioral coding (see Table 5). We could see the child’s face for the whole session in 35 videos (73%), and in seven videos (15%), we could not see the child’s face for only a little of the video (less than 25% of duration). For videos where parents fed their child, their hands did not cover the child’s mouth at all in 44 sessions (92%). 40 children (83%) were seated in one place and did not move around (were in the video frame) for the entire video, and six (13%) moved around a little bit.

**Table 5:** Frequencies (%) of video issues in naturalistic videoconference meal time observations.

	Not an issue	Little of the video	Half of the video	Most of the video	All of the video
Cannot see parent’s face	17 (35)	8 (17)	3 (6)	5 (10)	15 (31)
Cannot see child’s face	35 (73)	7 (15)	2 (4)	4 (8)	0
Parent’s hand comes in front of child’s mouth	44 (92)	1 (2)	1 (2)	2 (4)	0
Lighting issues (e.g., video too dark)	37 (77)	1 (2)	0	0	0
Audio not clear	43 (90)	4 (8)	1 (2)	0	0
Child moving around	40 (83)	6 (13)	1 (2)	1 (2)	0
Bilingual/Not in English	44 (92)	0	1 (2)	1 (2)	2 (4)
Internet connectivity issues	41 (85)	7 (15)	0	0	0

Percentages rounded to nearest whole number. For the lighting issues category, for 10 videos (21%), the video was coded as “brightness is not great but can still see food/child.”

Parent visibility and language use. The data were mixed with regard to parents being in the frame. In 17 (35%) videos, the parents were in the frame the whole time, and in 15 (31%) videos, the parents were not in the frame at all. However, of the 20 videos in which the parent was not in the frame for most or all of the video, we could hear them talking in 18 (90%) videos. Parents spoke in English to their child in 44 videos (92%) and did not speak in English at all in two videos (4%).

Food visibility. For 26 (51%) of the children, we could see their food directly, for 23 (45%) children, we could see their eating set up but not the food directly unless it was picked up, and in two (4%) sessions, the view of the food was obstructed. Of the 12 feeding sessions that included bottle feeds, the children’s mouths were blocked by the bottle for most or all of the video in seven (58%) sessions.

General visibility and connectivity. In terms of visibility, 37 videos (77%) had good brightness for all of the video, followed by 10 videos (21%) where we could still see the feeding setup but the brightness was comparatively lower. The more challenging videos were the sibling studies when more than one child was eating together in the same session. Here, in all three of these sessions, we could not see individual children for most or all of the session which would interfere with food bites or individual eating behavior coding.

We also wanted to capture disruptions regarding to Internet connectivity. In 43 videos (90%), the audio was clear for the entire video, and in 41 videos (85%) there were no Internet connectivity issues. In seven videos (15%), Internet connectivity issues existed for a little (less than 25%) of the duration, which indicates brief freezing frames in the recording. In none of the videos was Internet connectivity an issue for the entire video (i.e., the family did not freeze completely, or we did not have to restart/cancel the session).

### Discussion

This feasibility study revealed that, for the most part, observational meal recordings garnered through synchronous videoconference sessions yield codable data. Specifically, researchers can view the child’s face, feeding setup, and food intake clearly, with reasonable audio and video quality and the child being seated in one place (i.e., not moving in and out of frame frequently). Although parents themselves were not present in these videos all the time, parent talk was recorded for subsequent coding (e.g., for researchers interested in parental prompts or other types of verbal engagement during meals). One potential reason why parents were not in the frame is because we did not explicitly tell them to be there. Parents interpreted our instructions differently, and hence, they were mixed in terms of who was visible in the frame (just the child or the parent and child), especially when the child self-fed. Similarly, we did not instruct parents as to what type of foods to feed their child, so some parents mentioned that they made their child’s most liked food to ensure they have a smooth session with us, while others tried an unfamiliar food as they believed it would be interesting for us. However, whether or not this variability would count as an “issue” for researchers depends completely on their research questions and can be solved through live feedback from the researcher to the parent, a topic we return to in the General Discussion.

As observed, 15 sessions were family meals, where parents and other family members could be seen eating along with the child in these videos. The presence of family and companions facilitates greater food intake during mealtimes (De Castro, 1994). Moreover, seeing adults or peers socially modeling eating increases children’s food intake (see Cruwys et al., 2015 for review). In this way, mealtime observations at home could offer the opportunity to study such social influences on food intake. However, this was not the focus of the present research as we aimed at assessing the feasibility of collecting data on children’s individual eating. Moreover, we found that in the videos that had more than one child in the frame, the data quality was reduced as all children were not always in view. Here, the extent of the issue is also dependent on the device used by parents to call into the session and how far away the device was placed from the children. If parents call in from their tablet or mobile phone, then their camera view is narrower. If parents physically move the camera from one child to another to correct for this narrow view and attempt to capture both children, it is actually more difficult to extract any data (as each child is only visible for some of the session), compared to focusing on only one child (which means losing one child’s data but having full data from another). If parents call in from a laptop device and place the laptop further away from the children to get a wider frame of the feeding setup, further distance reduces the ability to clearly view the food and child food bites. Hence, depending on one’s research question, the presence (and absence) of other family members can be facilitated in such a setup that occurs at home.

Additionally, compared to solid food sessions, a majority of the bottle feeds obstructed the view of the child’s mouth in the video recordings which would be challenging to code sucks. Therefore, it is critical for researchers to consider the type of data they hope to obtain, test out their videoconferences on multiple types of devices, and develop specific instructions to walk through with parents to capture the angles and information needed.

In addition to food bites, in Method 2 we describe the use of synchronous videoconference sessions to assess child food ratings and preferences.

## Method 2

### Asking Children Questions About Food

Apart from measuring actual food intake, another method of assessing children’s eating behavior is eliciting their food ratings or beliefs about foods (e.g., DeJesus et al., 2019b; Echelbarger et al., 2020). In this section, we highlight online studies conducted during the COVID-19 pandemic that have assessed children’s preferences and predictions as a plausible method for examining children’s opinions about foods using synchronous videoconference sessions. Specifically, we briefly describe the methods, exclusion criteria, and attrition across studies.

### Participants

We started data collection *via* synchronous videoconference sessions with children aged 3 to 12 years in May 2020 and have collected data from 192 children to date. We excluded 11 children’s data (detailed under “Results”), which yielded a usable sample of 181 children (98 female). In addition, 10 parents scheduled an appointment but their child(ren) did not ultimately participate in the study (two parents completed the consent form but did not attend the appointment, seven parents did not complete the consent form nor attend the appointment, and one parent chose not to participate after learning that they would need to log in using video). Collapsing across studies, our participants identify as 13 (7%) Latinx and 104 (57%) Caucasian/White (see Table 1). Across studies, we follow a similar recruitment protocol to Method 1 (i.e., families are recruited *via* our volunteer database, social media advertising, and Children Helping Science). Parents were emailed the consent forms specific to their child’s study in advance of the synchronous videoconference session. After the study, children were emailed a certificate and their choice of prize pack (an activity book of do-at-home science experiments, coloring sheets, word puzzles, recipes, or mazes) for participating.

### Screen-Sharing Check Procedure

**Figure 1:**
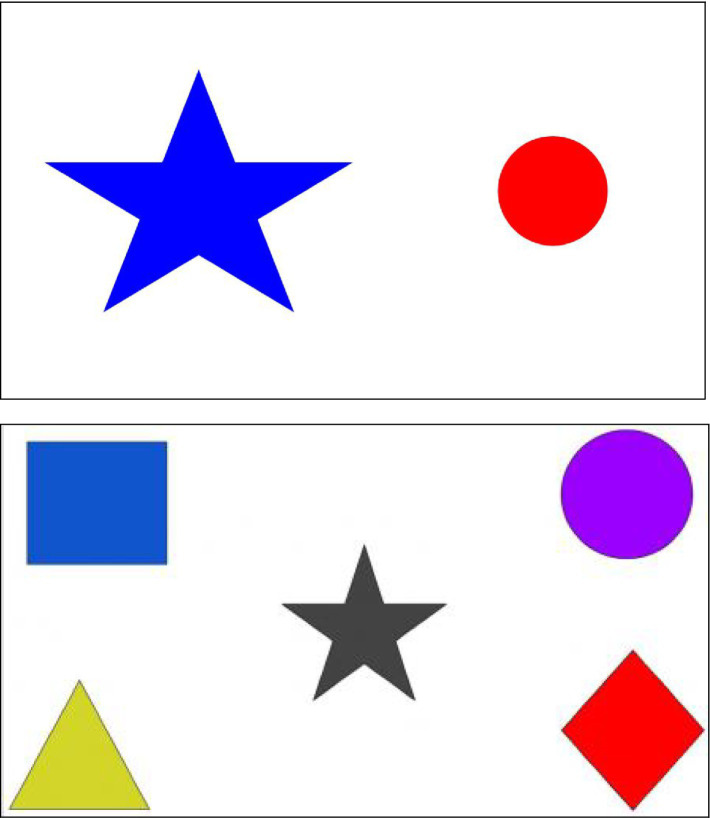
Images shown to children (top: under 7 years; bottom: 7 years and older).

In these studies, researchers shared their screen with participants. To ensure that children could see the researcher and the study images, participants first completed a screen check. For children younger than 7 years of age, after sharing their screen, the researcher made a thumbs-up sign and asked children if they could “do what I’m doing with my hand.” Then, children saw a picture of a blue star and a red circle (see Figure 1, top) and were asked to name the color of each shape. Children were asked which shape was bigger if they could not name the colors (e.g., one parent reported that their child was colorblind). For studies of children age 7 years and older, the researcher first held up three fingers and asked the child “how many fingers am I holding up?” Next, they asked the child to hold up two fingers. Finally, an image with five shapes was shown, and the child was asked the color of the rectangle and diamond. If they could not name the colors, the child was asked how many shapes they saw (see Figure 1, bottom).

### General Study Procedure

After the screen-sharing check, across research questions our studies involved showing children pictures of people and/or foods. Some studies included short stories about characters featured in the studies. Then, we asked children questions about these pictures or stories. For example, in one study, we showed children pictures about foods from different cultures, asked them their opinions of each of those foods, and who they think would be more likely to bring that food to school from an array of faces (Venkatesh and DeJesus, 2021). In another study, we showed children stories about characters who were sick and asked them to make predictions about disease transmission (DeJesus et al., in press).

### Results

Among younger children, all children passed the thumbs-up check and all passed a version of the shape check (92 passed the color check and eight passed the size comparison check). Among older children, all children passed the holding-up fingers check, and all passed the shape check (one child only answered the color of the diamond).

Across our studies, our *a priori* exclusion criteria were as follows:

(1) The child cannot see the researcher’s screen or experienced Internet connectivity issues (*n* = 1).
(2) The child asks to stop the study or walks away from the screen without intention of returning to the study (*n* = 4).
(3) The child observes their sibling participate before them or their sibling interferes with the study (*n* = 1).
(4) We do not receive the parent online consent form (*n* = 1).
(5) The parent interferes with the study (*n* = 3), and
(6) The parent signed up, but child was not of the correct age for the study (*n* = 1).

Note that parent interference was defined by a parent suggesting an answer or commenting on the child’s answer (such as “you like taking sandwiches to school!”). Responses were not excluded if the parent reminded the child to answer the researcher’s questions but without suggesting what the answer should be (such as “look, she is asking you a question!”); directing the child’s attention back to the researcher was especially helpful for studies with younger children (3- and 4-year-olds).

### Discussion

We had low rates of exclusion in studies of children’s preferences and predictions *via* synchronous videoconference sessions. We excluded 11 children and retained data for 181 participants (94%). From our experience as researchers, children’s ability to complete the session and share their opinions and preferences seemed comparable to in-person studies that are similar in format to the method described here. In line with our subjective experience, in a study that compared children’s thinking about disease transmission in person before the pandemic and on Zoom during the pandemic, we found little difference in children’s responses across time and platform (DeJesus et al., in press). Although we had few exclusions in these studies, anecdotally, studies that involved telling stories to children and asking them follow-up questions were especially challenging for children younger than age 4. We return to this issue in the General Discussion.

## General Discussion

In this paper, we have illustrated two ways to study children’s eating behavior at home using synchronous videoconference sessions. In the first method, we highlight a feasibility study in which we remotely observed meals and snacks at home with children under 3 years of age. Our analyses reveal that such designs yield video data that can be used for behavioral coding projects, based on the clear view of the feeding setup, child’s face, and parent–child engagement in most videos. The main benefit of this paradigm is its ecological validity. Studies of eating behavior that are primarily conducted in settings outside the child’s home, such as in the laboratory or in structured observations at schools or community centers that resemble in-lab studies (Fernandez et al., 2018; DeJesus et al., 2018b), are valuable but may not be representative of the child’s typical food environment. Our observational study which tested children at home provides a method to study children’s eating behavior in a familiar environment. Children ate at their typical seats using cutlery and utensils they were familiar with, which may be especially useful to study children’s reactions to familiar vs. unfamiliar foods (Moding et al., 2014; DeCosta et al., 2017). Testing children at home removes the additional variable of the unfamiliarity of an in-lab setting.

From a logistical perspective, studying children’s eating behavior at home reduces the personnel and setup required for in-person lab feeding studies. First, in-person lab studies require a laboratory space, ideally with parking and access to public transportation, which researchers may not have available to them. Then to offer foods in an in-person lab study, researchers face additional challenges, including acquiring foods (especially for researchers interested in studying children’s willingness to eat vegetables and other perishable foods) and avoiding common allergens. Moreover, laboratory studies typically standardize foods across participants, yet a food that is unfamiliar to one child might be familiar to another. Thus, while researchers might lose control over the standardization of the foods and environments that are possible for in-lab studies, at-home observational studies give parents the option of choosing foods that are familiar or unfamiliar to their child. This approach also gives parents the flexibility to schedule the testing session according to the child’s current meal schedule (especially for infants when their mealtimes are more variable) without having to travel to another location. Even for observational studies of children’s eating behavior at home described previously, researchers face logistical hurdles in terms of making trips to families’ homes. This may require researchers to have access to transportation (e.g., to directly observe families or pick up and drop off recording equipment) and requires parents to be comfortable inviting researchers into their homes.

Another advantage of synchronous videoconference sessions is the option of giving live feedback to the parents. This feedback can serve multiple purposes. First, researchers can provide instructions to improve data quality. Synchronous videoconference sessions allow researchers to guide parents to ensure the camera is positioned accurately (compared to distributing video cameras for parents to use at home). Second, researchers can use this feedback to give parents specific instructions for an experimental manipulation. Although we chose not to give parents any specific instructions to make the session as easy as possible for parents and assess whether videoconference would be a suitable platform for research measuring children’s eating behavior, many types of specific instructions could be given to bring in some of the control of laboratory studies. For instance, researchers can tell parents what type of foods to feed their child, instruct parents with specific prompts (such as feed your child an unfamiliar food for 5 min), or provide standardized types and amounts of foods (e.g., through delivering foods directly to parents) depending on the research question at hand.

In Method 2, we were able to collect behavioral data from 3- to 12-year-old children on their ratings and predictions. We had low rates of exclusion across studies (we were able to retain 94% of participants), and children were able to see our pictures and hear us accurately, as indicated by the screen-share check questions. Such methods closely resemble food preference and rating studies conducted in the laboratory or other community settings (Rioux et al., 2016; DeJesus et al., 2019a; Echelbarger et al., 2020). Studies were run directly from Qualtrics, which reduced the extra step of running the study on another platform (such as Microsoft PowerPoint) and entering the data separately. Qualtrics is limited in its video storage capacity, so studies that include showing videos to participants require alternative presentation methods (e.g., embedding YouTube videos in Qualtrics or showing the video from another platform). None of the studies described here include videos, so we do not have data on potential exclusions due to insufficient connectivity to play videos (either from the researcher’s side or the participant’s side), which would be more prone to disruption. However, Method 2 appeared to be especially difficult for children younger than 4 years of age, especially without videos or detailed animations. Although we did not collect systematic data on this experience, anecdotally, it was much more difficult to complete synchronous videoconference sessions with children younger than age 4 (and even for some 4-year-old children) in terms of their understanding of their interaction with the researcher. For instance, some parents reported that their child might not fully understand that they were interacting with a real person.

### Limitations and Challenges

While the present research highlights the potential to use synchronous videoconference sessions to conduct research on children’s eating behavior, we interpret our claims with caution. This method limits the types of measurements that researchers can include in their data to those that can be seen or heard. Many studies that measure children’s eating behavior includes child body mass index (BMI) or infant weight-for-length z-scores as predictors or outcomes in their analyses (e.g., Bergmeier et al., 2015a; Lumeng et al., 2020; Ventura and Hupp, 2020), which cannot be measured directly in a videoconference session. One approach to estimating this data could be to use coding tools that just require still images from the videoconference sessions. For example, the Shapecoder tool was designed to provide a coding system for child BMI and has both high inter-rater reliability and is correlated with child BMI measurements (Park et al., 2018). Researchers interested in using this tool may need multiple unobscured angles of the child (i.e., not blocked by a table). A similar tool is not currently available for infants, but researchers could consider asking parents for their child’s measurements at their last pediatrician visit. Although parents tend to underestimate their child’s weight (Eckstein et al., 2006; Lundahl et al., 2014), parents may have access to this data electronically through their healthcare provider, or parents of infants could have better recollection for their infants’ measurements due to more frequent pediatrician visits. We did not attempt to study the feasibility of collecting height and weight measurements in these studies, but it is possible that some estimate could be attainable.

Importantly, our participant demographics represent homogenous families who were majority White, highly educated, and from higher income brackets. We relied on the platform Children Helping Science for recruitment, which is frequented by parents who are researchers/faculty themselves and may be familiar with online research and the challenges of continuing research programs during the pandemic. The vast majority (85%) of our meal recordings did not have substantial Internet connectivity issues, and we excluded only one of our verbal preference studies for network connectivity disruptions. Our sample’s higher socioeconomic status is suggestive of their access to stable Internet connections and technology (e.g., updated and reliable smartphones, tablets, or computers) which enabled them to participate in such studies. Although some note remote online testing as an opportunity to include families from diverse backgrounds in child development research (e.g., Rhodes et al., 2020), the digital divide may further exclude participants from minority and lower socioeconomic backgrounds who not only have limited access to the Internet connectivity required for online data collection, but who are also faced with economic and childcare inequalities and have been most impacted by the COVID-19 pandemic (Lourenco and Tasimi, 2020). Especially pertinent to food-related research, such populations are also more likely to encounter food insecurity and rely on food assistance programs during the pandemic (Gassman-Pines and Gennetian, 2020). Thus, while synchronous videoconference sessions allowed us to interact with families who were diverse geographically (rather than being limited to our local area), our sample is restricted in its racial/ethnic and socioeconomic diversity. Our feasibility findings can only be generalized to families who are from the similar social and economic backgrounds as in our sample. Similar concerns surrounding access also apply to our research team – our research assistants who previously assisted with in-person lab studies also needed sufficient technology and private spaces to assist with research studies by videoconference, potentially leading to inequities in access to high impact teaching practices, such as participating in hands-on research activities (e.g., Kuh, 2008).

This videoconference method required basic parental literacy of video-chat applications (i.e., being able to be seen and heard on video) that we also shared *via* a guide with them. We did not experience issues with the setup of the call in any of our sessions. While parents might be more familiar with certain video chat applications (such as FaceTime), Zoom, and WebEx provide the option to record to the device (and not the cloud) which enhances the safety of the recordings and provides a standard option across families (e.g., families that did not have Apple devices did not have access to FaceTime when we began the study). Anecdotally, with the ubiquitous use of Zoom during the year of the pandemic, parents and children were more familiar and comfortable with the application compared to when we initially used WebEx for data collection. Nonetheless, more research is needed to better describe children’s understanding of interactions by video and their views on being videotaped, which may vary across children. Outside of our specific research questions, even young children are able to have positive interactions that build relational connections on video (McClure and Barr, 2017; McClure et al., 2021), though this may not fully generalize to conversations with unfamiliar researchers they are meeting for one session. At the same time, while children’s understanding of some aspects of digital privacy is developing (Gelman et al., 2018; Sun et al., 2021), more research is needed to better understand children’s beliefs, knowledge, and preferences in this area.

### Recommendations and Future Directions

Based on our experiences of conducting the present research, we have the following recommendations to researchers who seek to use synchronous videoconference sessions to study children’s eating behavior:

(1) Closely consider what data you hope to attain and develop instructions to ensure that behavior is visible on the video.
(2) Plan on changing the requirements of those instructions based on the device the parent logs-in from. Different devices (smartphones, tablets, and laptops) contain varying ranges of view for a video frame, so consider asking the parent what device they are using and share instructions for positioning/lighting accordingly.
(3) Studies that ask children to follow stories may not be accessible to children under the age of 4 or 5. There are many potentially interesting questions to ask with 2- to 4-year-olds that primarily observe children’s behavior or enlist parents as the experimenter (rather than relying on their ability to interact on videoconference with an unfamiliar person).
(4) Consider creating a demonstration version of your study in case of serious Internet connectivity issues. For instance, if families do not have sufficient Internet connectivity to pass the screen-sharing check or turn on their video, it will be helpful to have some open-ended questions for the child or parent to answer. Such demonstrations may be familiar to researchers who work in museums or other community settings, where it is often useful to have a related demonstration activity for children whose parent/guardian is not present or would prefer not to sign consent documents. This demonstration would still give families the opportunity to engage with the research process and discuss their experiences with the researcher.
(5) Make use of the live session to ensure parents fill out the online consent form (if they have not already) before you start the session with the child and to clarify data entered in the consent form that may contain typos (for example, birthdates).
(6) If possible, have Internet hot spots and additional technology available for members of the research team to check out. Note that hot spots may not improve Internet access in low coverage areas.
(7) Target multiple social media and online platforms for recruitment. In addition, consider physical advertisements in your community. This may raise the profile of your research to families who may not be as reachable using social media.

In addition to these recommendations, there are several topics that we view as possible to study using remote methods but have not yet pursued. We review two here in more detail. First, before infants begin eating solid foods, food intake is often measured using sucking behavior. Although sucking behavior can be coded from video recordings (Lumeng et al., 2007), this may be a difficult task to complete over videoconference. Based on our small number of bottle feedings, detailed instructions for parents on camera placement would be needed to achieve the close and unobstructed view of the infant’s face that is needed for video coding. Alternatively, devices, such as the Neonur, can record infants’ continuous negative sucking pressure and sucking bursts, or clusters of sucks that occur within less than 2 s between each suck (Lumeng et al., 2020); however, such devices would need to be exchanged with parents (which may be challenging with limited interaction and available team members). Second, digital imaging can be used to identify foods on a plate and measure food intake. In an intervention that explored whether involving children in making foods would increase their willingness to try new foods, researchers assessed children’s snack choices after the intervention by comparing pictures of their plates before and after intake (Allirot et al., 2016). Similarly, the contents and nutritional quality of children’s packed lunches were coded from photographs of the participating children’s lunchbox contents before children ate lunch (Sutter et al., 2019). Researchers can also measure the healthfulness of meals consumed through “plate analysis” or examining what types of foods are on children’s plates, for instance using the Healthy Meal Index (Kasper et al., 2016). Parents could share pictures of children’s plates/meals for analysis by researchers, an even smaller commitment of time and technology compared to a videoconference study.

## Conclusion

This paper has illustrated how synchronous videoconference sessions can used to study children’s feeding behaviors, adding to existing work that use these designs to examine children’s cognition, emotion, language, and social development. Using these sessions to observe meals provides ecological validity for children’s eating behaviors and allow for live researcher feedback. Various measures can be collected through these methods, such as bites or pieces eaten, meal characteristics (such as the feeding setup or whether it is a family meal), and parent–child talk during meals. While researchers may have to compromise the standardization of foods and environment that laboratory settings offer, we gain the generalizability of findings and increased participant scheduling flexibility. Moreover, researchers can use videoconference sessions to verbally assess children’s beliefs and preferences of different foods. While we are grateful for platforms, such as Children Helping Science, that have significantly enabled our laboratory’s continued data collection during the pandemic, we are also mindful of the representation in our sample. Ultimately, there is much to be gleaned about children’s eating behaviors and synchronous videoconference sessions can be a useful tool for researchers interested in connecting with families at home.

## Data Availability Statement

The datasets presented in this study can be found in online repositories. The names of the repository/repositories and accession number(s) can be found at: Open Science Framework, https://osf.io/rhmuq/

## Ethics Statement

The studies involving human participants were reviewed and approved by UNC Greensboro Institutional Review Board. Written informed consent to participate in this study was provided by the participants’ legal guardian/next of kin.

## Author Contributions

SV and JD conceptualized the studies, collected the data for Methods 1 and 2, and contributed to writing the manuscript. SV directed the video issues coding and analyzed the data in Method 1. All authors contributed to the article and approved the submitted version.

### Conflict of Interest

The authors declare that the research was conducted in the absence of any commercial or financial relationships that could be construed as a potential conflict of interest.

## Footnotes

^1^https://osf.io/rhmuq/

^2^https://www.cdc.gov/ncbddd/actearly/amazingme.html
